# Dietary Cholest-4-en-3-one, a Cholesterol Metabolite of Gut Microbiota, Alleviates Hyperlipidemia, Hepatic Cholesterol Accumulation, and Hyperinsulinemia in Obese, Diabetic *db/db* Mice

**DOI:** 10.3390/metabo14060321

**Published:** 2024-06-03

**Authors:** Mina Higuchi, Mai Okumura, Sarasa Mitsuta, Bungo Shirouchi

**Affiliations:** 1 Nutrition Science Course, Division of Human Health Science, Graduate School of Regional Design and Creation, University of Nagasaki, Siebold, 1-1-1 Manabino, Nagayo-cho, Nishi-Sonogi-gun, Nagasaki 851-2195, Japan; 2 Department of Nutrition Science, Faculty of Nursing and Nutrition, University of Nagasaki, Siebold, 1-1-1 Manabino, Nagayo-cho, Nishi-Sonogi-gun, Nagasaki 851-2195, Japan

**Keywords:** cholest-4-en-3-one, 4-cholestenone, hyperlipidemia, hyperinsulinemia, *db/db* mice

## Abstract

Previous studies have shown that dietary cholest-4-en-3-one (4-cholestenone, 4-STN) exerts anti-obesity and lipid-lowering effects in mice. However, its underlying mechanisms are not fully understood. In the present study, we evaluated whether 4-STN supplementation would protect obese diabetic *db/db* mice from obesity-related metabolic disorders. After four weeks of feeding of a 0.25% 4-STN-containing diet, dietary 4-STN was found to have significantly alleviated hyperlipidemia, hepatic cholesterol accumulation, and hyperinsulinemia; however, the effect was not sufficient to improve hepatic triglyceride accumulation or obesity. Further analysis demonstrated that dietary 4-STN significantly increased the content of free fatty acids and neutral steroids in the feces of *db/db* mice, indicating that the alleviation of hyperlipidemia by 4-STN was due to an increase in lipid excretion. In addition, dietary 4-STN significantly reduced the levels of desmosterol, a cholesterol precursor, in the plasma but not in the liver, suggesting that normalization of cholesterol metabolism by 4-STN is partly attributable to the suppression of cholesterol synthesis in extrahepatic tissues. In addition, dietary 4-STN increased the plasma and hepatic levels of 4-STN metabolites cholestanol (5α-cholestan-3β-ol) and coprostanol (5β-cholestan-3β-ol). Our results show that dietary 4-STN alleviates obesity-related metabolic disorders, such as hyperlipidemia, hepatic cholesterol accumulation, and hyperinsulinemia, in *db/db* mice.

## 1. Introduction

Steroids are natural or synthetic organic compounds that are characterized by a molecular structure of 17 carbon atoms arranged in four rings [1]. The steroid group plays a structural role in cell membranes and contributes to physiological functions as a regulator of several important metabolic pathways as secondary messengers and hormones. For example, cortisol and synthetic cortisol-like compounds, termed as corticosteroids, help regulate blood pressure, immune function, and anti-inflammatory processes [2,3]. Moreover, phytosterols and phytostanols (e.g., β-sitosterol, campesterol, sitostanol, campestanol, and saringosterol) have been reported to exert biological effects, including lowering blood low-density lipoprotein cholesterol (LDL-C) levels, improving cognition function, and alleviating the pathology of Alzheimer’s disease [4,5,6,7]. A growing body of evidence suggests that structural analogs/isomers of steroids are generated by various oxidation and in vivo metabolic reactions, and that the physiological functions of steroid analogs/isomers differ depending on the presence or absence of carbon–carbon double-bonds and their position as well as the type of side chain [8,9].

**Figure 1:**
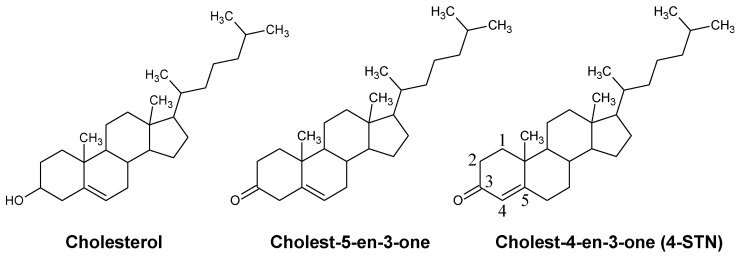
Chemical structures of cholesterol, cholest-5-en-3-one, and cholest-4-en-3-one (4-cholestenone, 4-STN).

Cholest-4-en-3-one (4-cholestenone, 4-STN) is a steroid that has an oxo group at the carbon-3 (3-oxo) position and a double bond at the carbon-4 position (Figure 1). 4-STN oxime, namely, olesoxime (TRO19622), is one of the mitochondrial-targeted neuroprotective compounds being studied as a drug for amyotrophic lateral sclerosis (ALS) and spinal muscular atrophy (SMA) [10]. 4-STN is a metabolic intermediate generated during the conversion of cholesterol to coprostanol by gut microbiota [11]. Generally, 4-STN is generated in the large intestine, which is the primary site of gut microbiota; therefore, it does not pass through the small intestine, which is the organ responsible for absorbing dietary lipids, including cholesterol. Therefore, the physiological functions of dietary 4-STN are thought to be different from those of dietary cholesterol. Previous studies have shown that dietary 4-STN reduces serum cholesterol levels in rodents by suppressing endogenous cholesterol biosynthesis [12,13] and abdominal fat deposition [14,15]. However, the mechanism underlying the action of dietary 4-STN remains unclear. Interestingly, a recent study showed that dietary cholest-5-en-3-one (Figure 1), a metabolic precursor of 4-STN, alleviated hyperglycemia and hyperinsulinemia, and reduced serum triglyceride levels in obese diabetic *db/db* mice [16]. Therefore, dietary 4-STN, a structural analog of cholest-5-en-3-one, is also hypothesized to exert these beneficial effects.

To gain insights into the physiological function of dietary 4-STN, in the present study, we investigated the effects of dietary 4-STN on the development of obesity-related metabolic disorders in obese diabetic *db/db* mice. This strain of mice has hyperphagia due to a missense mutation in the leptin receptor gene and develop multiple metabolic and hormonal disorders, including hepatic lipid accumulation (i.e., nonalcoholic fatty liver disease) and type 2 diabetes, which share many features with metabolic syndrome in humans [17,18].

## 2. Materials and methods

### 2.1. Animals and Diets

All experiments were conducted in accordance with the Guidelines for Animal Experiments of University of Nagasaki, Siebold, and Law No. 105 and Notification No. 6 of the government of Japan. The animal protocol used in this study was approved by the Institutional Review Board of University of Nagasaki, Siebold (authorization no. R03-19). 

Five-week-old male C57BL/6J (C57BL/6JJcl) and *db/db* (BKS.Cg-+*Lepr^db^*/+*Lepr^db^*/Jcl) mice were purchased from CLEA Japan, Inc. (Osaka, Japan). The mice were housed individually in plastic cages in a temperature-controlled room at 22 ± 1 °C with 55 ± 5% humidity, under a 12 h light/dark cycle. The experimental diets were prepared according to the AIN-76 formula [19] with several modifications (Table 1). 4-STN (purity > 97%) was purchased from Santa Cruz Biotechnology, Inc. (Dallas, TX, USA). *db/db* mice were assigned to two groups (*n* = 6/group) that were fed one of the following diets (Table 1): a control diet containing 7% soybean oil and 0.1% cholesterol (CON group) or control diet supplemented with 0.25% 4-STN at the expense of sucrose (4-STN group). C57BL/6J mice (*n* = 6), which are the progenitors of *db/db* mice, were fed a control diet (NOR group). The mice were allowed free access to the diets using Rodent CAFE (KBT Oriental, Saga, Japan) and to water for four weeks. Feces were collected for six days prior to the end of the experiment. At the end of the feeding period, the mice were sacrificed by exsanguination from the heart under isoflurane anesthesia after a 9 h starvation period. Blood sample was immediately mixed with EDTA-2Na (final concentration 1.2 mg/mL) on ice. Plasma was collected by centrifugation at 1200× *g* for 15 min, at 4 °C. The liver, pancreas, abdominal (epididymal, perirenal, and mesenteric) white adipose tissue (WAT), brown adipose tissue (BAT), and soleus muscles were excised and weighed within 5 h. The collected samples were stored at –80 °C until further analysis.

### 2.2. Respiratory Gas Analysis

After 3 weeks of feeding with the experimental diets, each mouse was placed in an acrylic metabolic chamber (120 × 150 × 240 mm) for 24 h to measure VO_2_ (oxygen exhaustion) and VCO_2_ (carbon dioxide emission). During respiratory gas analysis, the mice were pair-fed and had free access to water. The system consisted of 16 acrylic metabolic chambers, a mass spectrometer (ARCO-2000; ARCO SYSTEM, Inc., Chiba, Japan), a gas sampler (ARCO-2000-GS-16; ARCO SYSTEM, Inc.), and software (ARCO-2000-RAT; ARCO SYSTEM, Inc.). Room air was pumped into the chambers at a rate of 0.3 L/min. Expired air was passed through a cellulose acetate membrane filter (LABODISC^®^ 50CP020AN, pore size 0.20 mm, Toyo Roshi Kaisha, Ltd., Tokyo, Japan) and then directed to the mass spectrometer. Air from each chamber was sampled every 5 min, and the resulting data were recorded on a spreadsheet. Carbohydrate and fat oxidation, and energy expenditure were calculated using the following formulas:

Carbohydrate oxidation = 4.51 × VCO_2_ − 3.18 × VO_2_

Fat oxidation = 1.67 × (VO_2_ − VCO_2_)

Energy expenditure = 3.816 × VO_2_ + 1.231 × VCO_2_

### 2.3. Measurement of Plasma Biochemical Parameters

Plasma levels of triglycerides, total cholesterol, phospholipids, free fatty acids, glucose, and alanine transaminase (ALT) were measured using commercial enzyme assay kits (Triglyceride E-test, Cholesterol E-test, Phospholipid C-test, non-esterified fatty acids [NEFA] C-test, Glucose CII-test, and Transaminase CII-test, respectively; FUJIFILM Wako Pure Chemical Co., Osaka, Japan). The plasma high-density lipoprotein (HDL) fraction was separated as described previously [20]. Triglyceride and cholesterol levels in the plasma HDL fraction were measured using commercial enzyme kits (triglyceride and cholesterol E-tests; FUJIFILM Wako Pure Chemical Co.). Plasma non-HDL triglyceride levels were calculated as the difference between triglyceride and HDL triglyceride levels. Plasma levels of non-HDL cholesterol were calculated as the difference between total cholesterol and HDL cholesterol levels. Plasma levels of adiponectin, insulin, and leptin were measured using commercial mouse ELISA kits (mouse/rat adiponectin ELISA kit, Otsuka Pharmaceutical, Tokyo, Japan; LBIS mouse insulin ELISA kit, Shibayagi, Gunma, Japan; Mouse/Rat Leptin ELISA kit, Morinaga Institute of Biological Science, Inc., Kanagawa, Japan). The homeostasis model assessment of insulin resistance (HOMA-IR) was calculated using the following equation to assess insulin resistance: HOMA-IR = (fasting plasma glucose level (mg/dL) × fasting plasma insulin level (µU/mL)/405).

### 2.4. Measurement of Triglyceride, Cholesterol, and Glycogen Contents in the Liver

Total lipids from the liver (0.1 g) were extracted using the Bligh and Dyer method with slight modifications, as described previously [21]. The extracted lipids were dissolved in 2-propanol and adjusted to a volume of 2.0 mL for subsequent measurements. Hepatic triglyceride, total cholesterol, and free cholesterol contents were measured using commercial enzyme kits (triglyceride, cholesterol, and free cholesterol E-tests, respectively; FUJIFILM Wako Pure Chemical Co.) [22]. Hepatic esterified cholesterol content was calculated as the difference between the total and free cholesterol content. The hepatic phospholipid content was measured according to the method described by Rouser et al. [23]. Hepatic glycogen content was measured according to the method described by Lo et al. [24].

### 2.5. Measurement of Triglyceride and Free Fatty Acids Contents in Feces

The collected feces were lyophilized for two days. The lyophilized feces were powdered using a food processor (TML162; Tescom Denki Co., Ltd., Tokyo, Japan). Total fecal lipids were extracted as described by Jeejeebhoy et al. [25] with slight modifications. The extracted lipids were dissolved in 2-propanol for measurement. Fecal triglyceride and free fatty acid content were measured using commercial enzyme kits (Triglyceride E-test and NEFA C-test, respectively; FUJIFILM Wako Pure Chemical Co.). Fecal total bile acid content was measured according to the method described by Shirouchi et al. [26], with slight modifications. Fecal total bile acids were extracted with hot ethanol (70 °C, 60 min). The extracts were analyzed using a commercial enzyme kit (total bile acid test, FUJIFILM Wako Pure Chemical Co.). 

### 2.6. Measurement of Steroid Contents in the Liver, Plasma, and Feces

Cholesterol precursors, such as squalene and desmosterol, reflect cholesterol synthesis [27,28]. The levels of these cholesterol precursors and cholesterol metabolites, such as cholestanol and coprostanol in the plasma, liver, and feces, were measured by a gas chromatography–mass spectrometry (GC-MS) system using the Shimadzu GCMS-QP2010 Ultra (Shimadzu Corporation, Kyoto, Japan) equipped with an InertCap 5MS/NP capillary column (30 m × 0.25 mm i.d., 0.25 μm thickness, GL Sciences Inc, Tokyo, Japan) using 5α-cholestane (Cayman Chemical Company, Ann Harbor, MI, USA) as an internal standard. Briefly, 500 μL of hepatic lipid extraction, 25 μL of plasma, or 0.02 g of lyophilized and powdered feces was added to 2.5 μg of 5α-cholestane. The detailed sample preparation and analytical conditions of GC-MS were the same as described previously [29]. Peak identification of each steroid was performed by comparison of the retention time and mass spectra of authentic standards. Peaks with an S/N ratio of less than 3 were treated as the detection limit.

### 2.7. Measurement of mRNA Levels in the Liver and Epididymal WAT

Total RNA was extracted from frozen liver tissue soaked in RNA Save (Biological Industries Isarael Beit Haemek Ltd., Haemek, Israel) using RNAzol^®^ RT reagent (Molecular Research Center, Inc., Cincinnati, OH, USA) with 4-bromoanisole (Molecular Research Center, Inc.). Total RNA was extracted from epididymal WAT soaked in RNA Save using RNeasy^®^ Lipid Tissue Mini Kit (QIAGEN, Hilden, Germany). The detailed analytical conditions regarding RT-qPCR were the same as described previously [29]. Stable internal reference genes are crucial for RT-qPCR. In the present study, the expression of eight housekeeping genes (*Hprt1*, *Pgk1*, *Rpl13*, *Rpl32*, *Rplp0*, *Tbp*, *Ubc*, and *Ywhaz*) in the liver and epididymal WAT was evaluated. Relative mRNA levels were determined using the Pfaffl method [30] with *Tbp* as a housekeeping gene in epididymal WAT and *Ubc* as a housekeeping gene in the liver. In epididymal WAT, mRNA levels of genes involved in inflammatory response, such as *Ccl2* encoding monocyte chemoattractant protein (MCP)-1 and *Il6* encoding interleukin (IL)-6 (IL-6), and those involved in insulin signaling, such as *Irs1* encoding insulin receptor substrate (IRS) 1 and *Irs2* encoding IRS2, were measured. In the liver, mRNA levels of genes involved in inflammatory response (*Ccl2* and *Tnf* encoding tumor necrosis factor-alpha [TNF-α]), endoplasmic reticulum [ER] stress response (*Mapk8* encoding mitogen-activated protein kinase 8 [also known as JNK1] and *Xbp1* encoding X-box binding protein-1 [XBP-1]), insulin signaling (*Irs1*, *Irs2*, *Akt2* encoding protein kinase B2 [PKB2], and *Pik3ca* encoding phosphatidylinositol-4,5-bisphosphate 3-kinase catalytic subunit alpha [also known as PI3K]), and gluconeogenesis (*Foxo1* encoding forkhead box O1 [FOXO1] and *Pck1* encoding phosphoenolpyruvate carboxykinase 1 [PEPCK1]) were measured. The primer sequences used in this study are listed in Table S1.

### 2.8. Statistical Analysis

All values except for non-parametric data are expressed as the mean ± standard error of mean (SEM). Data from the NOR group were treated as reference data and were not used for statistical analysis. All data, except for those of the NOR group, were analyzed using the *F*-test to assess the equality of variance between the CON and 4-STN groups. Statistical analysis of parametric data with equal or unequal variances was performed using Student’s *t*-test or Welch’s *t*-test. Non-parametric data were expressed using box and whisker plots and were assessed using the Mann–Whitney’s *U*-test. Results with *p* < 0.05 were considered statistically significant, and those with 0.05 ≤ *p* < 0.1 were considered a tendency. Statistical analysis was performed using EZR, a graphical user interface of R (version 4.0.4) (The R Foundation for Statistical Computing, Vienna, Austria) [31]. 

## 3. Results

### 3.1. Effects of Dietary 4-Cholestenone on Nutrients Oxidation in db/db Mice

To examine the effects of dietary 4-STN on nutrient oxidation, respiratory gas analysis was performed with *db/db* mice after three weeks of feeding. Because the amount of food intake affects energy expenditure, the mice were fed a limited amount of the experimental diets (NOR group, 2.5 ± 0.1; CON group, 2.2 ± 0.3; 4-STN group, 2.3 ± 0.1 g, respectively) during respiratory gas analysis in the metabolic chambers. 4-STN feeding did not significantly alter total oxygen consumption (NOR group of C57BL/6J mice, 8.83 ± 0.11; CON group of *db/db* mice, 5.16 ± 0.45; 4-STN group of *db/db* mice, 5.05 ± 0.15 L/100 g B.W./day) and energy expenditure (NOR group of C57BL/6J mice, 43,260 ± 486; CON group of *db/db* mice, 25,018 ± 2227; 4-STN of *db/db* mice, 24,601 ± 657 cal/100 g B.W./day). In addition, 4-STN feeding did not significantly affect carbohydrate oxidation (NOR group of C57BL/6J mice, 7042 ± 102; CON group of *db/db* mice, 3072 ± 505; 4-STN group of *db/db* mice, 3440 ± 190 mg/100 g B.W./day) and fat oxidation (NOR group of C57BL/6J mice, 1804 ± 75; CON group of *db/db* mice, 1405 ± 167; 4-STN group of *db/db* mice, 1215 ± 145 mg/100 g B.W./day).

### 3.2. Effects of Dietary 4-Cholestenone on Morphometric Variables, and Biochemical Parameters in Plasma, the Liver, and Feces of db/db Mice

After four weeks of feeding with the experimental diets, there were no significant differences in the final body weight, food intake, food efficiency, organ and tissue weights, atherogenic index, or plasma levels of free fatty acids, ALT, adiponectin, and leptin in *db/db* mice between the CON and 4-STN groups (Table 2). Therefore, no side effects were observed in the present study due to the prolonged (four weeks) feeding of 4-STN at a low dose (0.25%).

**Figure 2:**
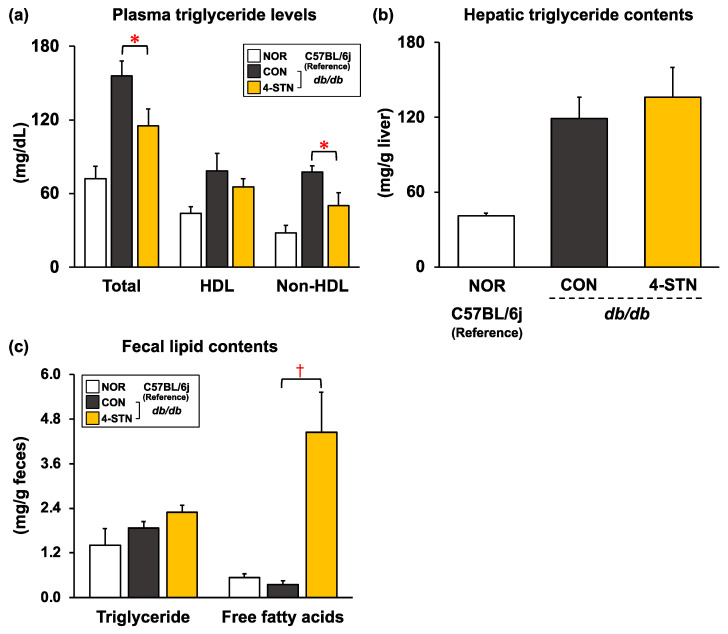
(**a**) Plasma triglyceride levels, (**b**) hepatic triglyceride contents, and (**c**) fecal contents of triglyceride and free fatty acids in C57BL/6J and *db/db* mice fed experimental diets for four weeks. Values are expressed as means ± SEM (*n* = 6/group). * *p* < 0.05 (vs CON group) analyzed by Student’s *t*-test. † *p* < 0.05 (vs. CON group) analyzed by Welch’s *t*-test. HDL: high-density lipoprotein; CON: *db/db* mice fed a control diet; 4-STN: *db/db* mice fed a 4-cholestenone-supplemented diet; NOR: C57BL/6J mice fed a control diet.

**Figure 3:**
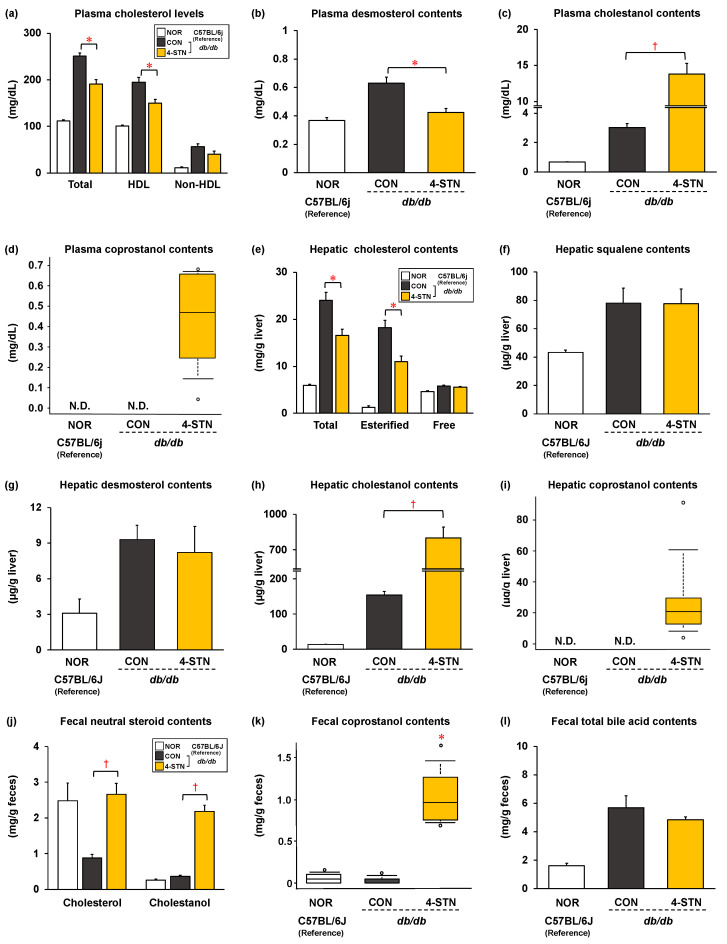
Plasma levels of (**a**) total, HDL, and non-HDL cholesterols, (**b**) desmosterol, (**c**) cholestanol, and (**d**) coprostanol; hepatic contents of (**e**) total, esterified, and free cholesterols, (**f**) squalene, (**g**) desmosterol, (**h**) cholestanol, and (**i**) coprostanol; and fecal contents of (**j**) neutral steroids (cholesterol and cholestanol), (**k**) coprostanol, and (**l**) total bile acids in C57BL/6J and *db/db* mice fed experimental diets for four weeks. Data except for (plasma, hepatic, and fecal coprostanol levels) are expressed as the mean ± SEM (*n* = 6/group). For plasma, hepatic, and fecal coprostanol levels, the box boundary closest to zero indicates the 25th percentile, a line within the box marks the median, and the box boundary farthest from zero indicates the 75th percentile. Whiskers (error bars) above and below the box indicate the 90th and 10th percentiles, respectively. In addition, outliers are graphed by dots. * *p* < 0.05 (vs. CON group) analyzed using Student’s *t*-test or Mann–Whitney’s *U*-test. † *p* < 0.05 (vs. CON group) analyzed using Welch’s *t*-test. N.D.: not detected. HDL: high-density lipoprotein; CON: *db/db* mice fed a control diet; 4-STN: *db/db* mice fed a 4-cholestenone-supplemented diet; NOR: C57BL/6J mice fed a control diet.

The CON group of *db/db* mice had obesity, hyperlipidemia, and hepatic lipid accumulation (fatty liver). As shown in Figure 2a, dietary 4-STN significantly decreased plasma triglyceride levels, which were associated with a significant decrease in plasma non-HDL triglyceride levels. Although no significant difference was observed in the hepatic and fecal triglyceride contents, fecal free fatty acid contents were markedly increased in 4-STN-fed mice (Figure 2b,c). In addition, dietary 4-STN significantly decreased plasma total cholesterol levels, which were associated with a significant decrease in plasma HDL cholesterol levels (Figure 3a). Hepatic total and esterified cholesterol contents were also significantly reduced in 4-STN-fed mice (Figure 3e). Although there was no significant difference in fecal acidic steroids (total bile acids) (Figure 3l), fecal neutral steroid (cholesterol, cholestanol, and coprostanol) contents were markedly increased in 4-STN fed mice (Figure 3j,k). Along with improvement in hyperlipidemia caused by dietary 4-STN, plasma phospholipid levels tended to be lower in mice that were fed the 4-STN diet than in those fed the control diet (*p* = 0.067) (Table 2).

**Figure 4:**
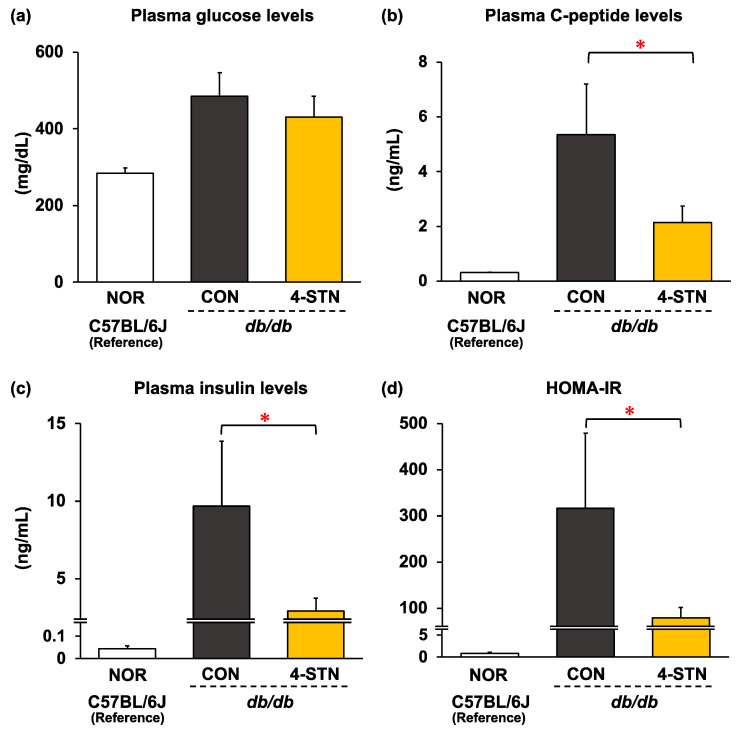
Plasma levels of (**a**) glucose, (**b**) C-peptide, and (**c**) insulin, and (**d**) HOMA-IR values in C57BL/6J and *db/db* mice fed experimental diets for four weeks. Data are expressed as the mean ± SEM (*n* = 6/group). * *p* < 0.05 (vs CON group) analyzed using Student’s *t*-test. CON: *db/db* mice fed a control diet; 4-STN: *db/db* mice fed a 4-cholestenone-supplemented diet; NOR: C57BL/6J mice fed a control diet.

The CON group also exhibited severe hyperinsulinemia. Mice fed the 4-STN diet had 11–21% lower plasma glucose levels (Figure 4a) and hepatic glycogen content (Table 2), with no significant difference when compared with those fed the control diet. On the other hand, plasma C-peptide and insulin levels were significantly decreased in the 4-STN group compared to that in the CON group (Figure 4b,c). The HOMA-IR value, an index of insulin resistance, was also significantly lower in the 4-STN group than that in the CON group (Figure 4d).

### 3.3. Effects of Dietary 4-Cholestenone on Cholesterol Precursor and Metabolite Levels in the Plasma and Liver of db/db Mice

Plasma desmosterol levels were significantly lower in the 4-STN group than that in the CON group (Figure 3b). In contrast, no significant differences were observed in hepatic squalene and desmosterol contents between the two groups (Figure 3f,g). Plasma and hepatic cholestanol levels were significantly higher in the 4-STN group than that in the CON group (Figure 3c,h). Coprostanol was detected only in the plasma and liver of 4-STN-fed mice (Figure 3d,i).

### 3.4. Effects of Dietary 4-STN on mRNA Levels in Epididymal WAT and the Liver of db/db Mice

Table 3 summarizes the effects of dietary 4-STN on the expression of several genes related to inflammatory responses and insulin signaling in epididymal WAT. Although *Irs1* mRNA levels tended to be lower in 4-STN-fed mice, no significant differences were observed in the expression of other genes. Table 3 also summarizes the effects of dietary 4-STN on the expression of several genes related to inflammatory response, endoplasmic reticulum (ER) stress, insulin signaling, and gluconeogenesis in the liver. *Pck1* mRNA levels were significantly higher in the 4-STN group than that in the CON group. No significant differences were observed in the expression of other genes.

## 4. Discussion

In the present study, we evaluated the effects of dietary 4-STN on the development of metabolic disorders in obese and diabetic *db/db* mice. We report that dietary 4-STN alleviated hyperlipidemia, hepatic cholesterol accumulation, and hyperinsulinemia in *db/db* mice.

Metabolic syndrome is a cluster of metabolic abnormalities, such as obesity, especially abdominal fat deposition, dyslipidemia, glucose intolerance, insulin resistance or hyperinsulinemia, and hypertension, leading to the development of type 2 diabetes and cardiovascular diseases [32]. As obesity can trigger metabolic syndrome, it is critical to maintain a healthy body weight, without fat deposition. Several food ingredients have been extensively studied and reported to exhibit anti-obesity activity [33,34,35,36,37]. Previous studies have shown that 17 months of feeding of a 0.5% 4-STN-containing diet significantly reduced body fat accumulation in CDF1 mice [14]. In the present study, four weeks of feeding of a 0.25% 4-STN-containing diet had no effect on energy expenditure and accumulation of abdominal fat (Table 2) in *db/db* mice. However, considering that dietary 4-STN significantly reduced plasma triglyceride levels and increased fecal free fatty acid content (Figure 2a,c), the anti-obesity effect of 4-STN needs to be examined further at different doses, for different feeding periods, and in different animal models.

Dyslipidemia is a well-known risk factor for complications, such as cardiovascular disorders and renal failure, emphasizing the importance of normalizing lipid metabolism in the diabetic context [38,39,40]. Suzuki reported a decreasing trend in the amounts of triglycerides and chylomicrons in the serum of CDF1 mice fed a 0.5% 4-STN-containing diet [14]. The results of the present study (Figure 2a,c) demonstrated that the triglyceride-lowering effect of 4-STN was mediated through inhibition of intestinal triglyceride absorption. Nagao et al. reported that dietary cholest-5-en-3-one, a metabolic precursor of 4-STN in the gut, reduced serum triglyceride levels in *db/db* mice [16]. Although phytosterols have recognized cholesterol-lowering effects, recent studies have focused on the triglyceride-lowering effects of phytosterols in animal models and human interventions [41]. Tomoyori et al. reported that dietary phytosterols reduced postprandial lymphatic triglyceride transport in thoracic duct-cannulated rats [42]. Ikeda et al. reported that dietary campest-5-en-3-one, an oxidized derivative of campesterol, significantly reduced serum triglyceride levels in rats [43]. Thus, our results are in line with these observations: several 3-oxo derivatives of cholesterol and their analogs may inhibit pancreatic lipase and/or affect micelle formation in the intestine, contributing to a triglyceride-lowering effect. As shown in Figure 3a, four weeks of feeding of a 0.25% 4-STN-containing diet significantly reduced the plasma levels of total cholesterol, HDL cholesterol, and hepatic cholesterol in *db/db* mice. To understand the mechanisms underlying the cholesterol-lowering action of 4-STN, we analyzed the levels of cholesterol precursor in the plasma and liver and fecal levels of cholesterol metabolites. Although the contents of hepatic cholesterol precursors and fecal total bile acids did not differ between the two groups (Figure 3f,g,l), plasma desmosterol levels were significantly lower (Figure 3b) and fecal neutral steroid content was significantly higher in the 4-STN group than that in the CON group (Figure 3j). Packie et al. reported that low doses (less than 0.5%) of 4-STN inhibited hepatic cholesterol synthesis and HMG-CoA reductase activity, whereas high doses (>3%) or prolonged 4-STN supplementation (7 days of feeding of a 1% 4-STN-containing diet) caused a rapid elevation in both hepatic cholesterol synthesis and HMG-CoA reductase activity to above-normal levels in the liver of several mouse strains [13]. Thus, we consider that prolonged (four weeks) feeding of 4-STN at a low dose (0.25%) obscured its inhibitory effect on hepatic cholesterol synthesis. However, plasma desmosterol levels were significantly lower in the 4-STN group than that in the CON group (Figure 3b). This is reflected in the suppression of cholesterol synthesis in the extrahepatic tissues, which led to a decrease in plasma HDL cholesterol levels. In addition, Wang et al. reported that dihydrocholesterol (5α-cholestan-3β-ol, cholestanol), a 4-STN metabolite, reduced cholesterol micellar solubility, leading to the inhibition of cholesterol absorption [44]. In the present study, the dihydrocholesterol (cholestanol) content was significantly increased in the feces of 4-STN-fed mice (Figure 3j). Therefore, in the intestine, 4-STN and its metabolites may affect micelle formation and inhibit lipid absorption. Taken together, these data suggest that the increase in fecal neutral steroid excretion and the suppression of cholesterol synthesis in extrahepatic tissues caused by 4-STN feeding contribute to the alleviation of hypercholesterolemia and hepatic cholesterol accumulation in *db/db* mice. From the perspective of the utilization and safety of 4-STN, further studies using hamsters and rabbits that have a lipoprotein metabolism similar to that of humans are needed to evaluate whether dietary 4-STN alleviates hypercholesterolemia without changing blood HDL cholesterol levels.

Excessive obesity leads to hyperinsulinemia and insulin resistance, which are major risk factors for type 2 diabetes [32]. It is well recognized that hyperinsulinemia results from resistance to insulin in glucose metabolism, leading to increased blood glucose levels, which stimulates pancreatic β-cells to release insulin to avoid severe hyperglycemia. In the present study, dietary 4-STN tended to decrease plasma glucose levels by 11% in *db/db* mice without statistical significance (Figure 4a) and significantly attenuated hyperinsulinemia and decreased HOMA-IR values (Figure 4c,d). C-peptide is secreted from pancreatic β-cells at an equimolar ratio to insulin and reflects endogenous insulin secretion more accurately than insulin because C-peptide, in contrast to insulin, is not extracted by the liver and other organs [45]. In the present study, dietary 4-STN significantly decreased plasma C-peptide levels in *db/db* mice (Figure 4b). In addition, dietary 4-STN tended to reduce hepatic glycogen content by 20% without statistical significance (Table 2) and significantly increased hepatic mRNA levels of *Pck1*, a critical enzyme in gluconeogenesis (Table 3). Altogether, the alleviation of hyperinsulinemia by dietary 4-STN may be attributed to the enhancement of glucose utilization and/or clearance, suggesting that hepatic gluconeogenesis was alternatively increased. A previous study showed that four weeks of feeding of a 0.25% cholest-5-en-3-one-containing diet alleviated hyperglycemia and hyperinsulinemia in *db/db* mice [16]. Another study also demonstrated that four weeks of feeding of 0.3–0.6% campest-5-en-3-one-containing diets exerted antidiabetic action in *db/db* mice and Zucker diabetic fatty rats [46,47]. Thus, our results are in line with these observations; several 3-oxo derivatives of cholesterol and their analogs may be dietary additives with antidiabetic action.

Chronic adipose tissue inflammation is involved in the development of insulin resistance [48]. According to a previous study, the alleviation of insulin resistance (hyperglycemia and hyperinsulinemia) by dietary cholest-5-en-3-one is attributable to a decrease in the production of inflammatory cytokines, such as *Ccl2* (MCP-1) and *Il6* (IL-6), in the adipose tissues [16]. Therefore, we evaluated the mRNA levels of genes involved in the inflammatory response, insulin signaling, and ER stress response in the epididymal WAT and liver. However, as shown in Table 3, no significant differences except for hepatic *Pck1* mRNA levels were observed in the present study. These discrepancies may be due to the addition of cholesterol to the experimental diets; the experimental diets used in the previous study [16] did not contain cholesterol, whereas our experimental diets contained 0.1% cholesterol. Excess cholesterol accumulation in multiple tissues and organs induces inflammation and ER stress, and plays an important role in the pathogenesis, development, and prognosis of multiple diseases [49]. Thus, increased inflammation caused by dietary cholesterol may mask the effects of 4-STN. However, considering that dietary 4-STN significantly reduced plasma and hepatic cholesterol levels, long-term 4-STN supplementation may alleviate inflammation.

Two gut microbiota metabolic pathways have been proposed for intestinal cholesterol metabolism [11]. Cholesterol is catabolized to cholest-5-en-3-one and 4-STN, followed by the production of 5β-cholestan-3-one (coprostanone), and finally the production of 5β-cholestan-3β-ol (coprostanol) [11]. Therefore, 4-STN is expected to be easily catabolized in the intestinal tract. However, it remains poorly understood whether dietary 4-STN is absorbed in its intact form and/or as metabolites, and then reaches the organs and tissues. To gain insight into the above, we measured the levels of 4-STN and its metabolites in the plasma and liver. 4-STN itself was not detectable because of the effect of fasting prior to dissection, whereas plasma and hepatic levels of cholestanol (5α-cholestan-3β-ol), a metabolite of 4-STN, increased significantly (Figure 3c,h). Moreover, coprostanol was detected in the plasma and liver of 4-STN-fed mice (Figure 3d,i). These results are consistent with a previous study that reported that the majority of intravenously injected C^14^-labeled 4-STN in rats is rapidly eliminated via feces and that C^14^-labeled 4-STN is rapidly metabolized in the liver, with cholestanol being the main product [50]. Our results suggest that 4-STN, which has a high turnover rate, and its metabolites (cholestanol and coprostanol) may be the active substances responsible for the physiological functions of dietary 4-STN.

There were several limitations to the present study. First, feeding of a 0.25% 4-STN-containing diet for four weeks significantly reduced plasma triglyceride levels and increased fecal free fatty acid contents (Figure 2a,c), but did not improve obesity in *db/db* mice. Therefore, the anti-obesity effect of 4-STN needs to be examined further at different doses, for different feeding periods using *db/db* mice, or in other animal models. Second, dietary 4-STN significantly attenuated hyperinsulinemia and decreased HOMA-IR values in *db/db* mice. However, dietary 4-STN did not affect the expression of genes related to ER stress, insulin signaling, and gluconeogenesis in the liver (Table 3). Thus, to better understand the mechanism by which dietary 4-STN attenuated hyperinsulinemia, future studies are needed to evaluate the expression of proteins related to ER stress, insulin signaling, and gluconeogenesis in the liver and muscle.

## 5. Conclusions

In summary, our results show that dietary 4-STN alleviates obesity-related metabolic disorders, such as hyperlipidemia, hepatic cholesterol accumulation, and hyperinsulinemia, in obese and diabetic *db/db* mice. To our knowledge, this is the first report to show that dietary 4-STN alleviates hyperinsulinemia, suggesting that 4-STN supplementation can potentially suppress the onset of type 2 diabetes. A comparison of the physiological functions of 4-STN and its metabolites (cholestanol and coprostanol) feeding will be of interest for future studies.

## Figures and Tables

**Table 1:** Composition of experimental diets in this study.

	Control Diet	4-STN Diet
Ingredients	(g/kg Diet)	
Sucrose	479	476.5
Casein	200	200
β-Cornstarch	150	150
Cellulose	50	50
Corn oil	70	70
4-STN	---	2.5
Mineral mixture (AIN-76)	35	35
Vitamin mixture (AIN-76)	10	10
DL-Methionine	3	3
Choline bitartrate	2	2
Cholesterol	1	1

**Table 2:** Effects of dietary 4-STN on morphometric variables in *db/db* mice.

	C57BL/6J	*db/db*	
	NOR Group	CON Group	4-STN Group
Initial B.W. (g)	21.0 ± 0.2	30.1 ± 0.4	30.0 ± 0.4
Final B.W. (g)	25.2 ± 0.3	36.2 ± 1.3	35.5 ± 0.9
Food intake (g/day)	2.97 ± 0.05	4.32 ± 0.19	4.29 ± 0.15
Food efficiency (mg B.W. gain/g food intake)			
	51.0 ± 3.7	48.3 ± 11.6	45.5 ± 6.5
Organ weight (g/100 g B.W.)			
Liver	4.06 ± 0.07	6.18 ± 0.41	5.95 ± 0.47
Pancreas	0.329 ± 0.020	0.253 ± 0.013	0.226 ± 0.010
Kidney	1.34 ± 0.02	1.08 ± 0.02	1.05 ± 0.04
Quadriceps muscle	1.52 ± 0.06	0.455 ± 0.014	0.478 ± 0.017
White adipose tissue weight (g/100 g B.W.)			
Perirenal	0.749 ± 0.072	2.16 ± 0.12	2.13 ± 0.09
Epididymal	2.00 ± 0.08	5.16 ± 0.13	4.86 ± 0.13
Mesenteric	0.969 ± 0.084	3.08 ± 0.09	3.25 ± 0.10
Brown adipose tissue weight (g/100 g B.W.)			
	0.545 ± 0.048	1.14 ± 0.13	1.25 ± 0.07
Feces weight (g/6 days)	1.60 ± 0.04	1.97 ± 0.25	2.10 ± 0.34
Plasma biochemical parameters			
Atherogenic index ^#^	0.115 ± 0.018	0.300 ± 0.050	0.274 ± 0.041
Phospholipid (mg/dL)	221 ± 6	323 ± 16	275 ± 17 ^(*p =* 0.067)^
FFAs (mmol/L)	0.930 ± 0.066	1.41 ± 0.11	1.55 ± 0.09
ALT (IU/L)	4.69 ± 0.36	25.4 ± 1.5	24.2 ± 3.1
Adiponectin (µg/mL)	16.4 ± 0.3	8.00 ± 0.42	7.02 ± 0.36
Leptin (ng/mL)	1.67 ± 0.33	42.4 ± 9.4	53.0 ± 8.6
Hepatic biochemical parameters (mg/g liver)			
Phospholipid	24.3 ± 0.6	20.2 ± 0.3	19.9 ± 1.4
Glycogen	5.03 ± 1.76	21.1 ± 3.4	16.7 ± 5.3

Values are mean ± SEM (*n* = 6/group). ALT: alanine aminotransferase; B.W.: body weight; FFA: free fatty acid. ^#^ Atherogenic index was calculated using the following formula: Non-HDL Chol/HDL Chol.

**Table 3:** Effects of dietary 4-STN on mRNA levels in epididymal WAT and the liver of *db/db* mice.

	C57BL/6J	*db/db*	
	NOR Group	CON Group	4-STN Group
Epididymal WAT	(arbitrary unit)		
Genes related to inflammatory response			
*Ccl2*	100 ± 18	165 ± 23	191 ± 18
*Il6*	100 ± 22	133 ± 19	189 ± 44
Genes related to insulin signaling			
*Irs1*	100 ± 6	40.5 ± 3.3	32.2 ± 1.0 ^(*p =* 0.056)^
*Irs2*	100 ± 9	36.6 ± 6.8	42.8 ± 6.0
Liver			
Genes related to inflammatory response			
*Tnf*	100 ± 14	167 ± 67	131 ± 30
*Ccl2*	100 ± 8	325 ± 110	335 ± 34
Genes related to ER stress response			
*Xbp1*	100 ± 7	145 ± 57	102 ± 23
*Mapk8*	100 ± 18	87.2 ± 10.2	112 ± 15
Genes related to insulin signaling and gluconeogenesis			
*Irs1*	100 ± 9	97.6 ± 16.6	86.9 ± 26.3
*Irs2*	100 ± 7	101 ± 26	92.1 ± 17.9
*Pik3ca*	100 ± 8	103 ± 10	114 ± 18
*Akt2*	100 ± 7	192 ± 54	139 ± 31
*Foxo1*	100 ± 11	156 ± 30	196 ± 25
*Pck1*	100 ± 14	76.1 ± 10.4	156 ± 29 †

CON: *db/db* mice fed a control diet; ER: endoplasmic reticulum; 4-STN: *db/db* mice fed a 4-cholestenone-supplemented diet; NOR: C57BL/6J mice fed a control diet; WAT: white adipose tissue. Values are means ± SEM (*n* = 6/group). † *p*< 0.05 (vs CON group) analyzed using Welch’s *t*-test.

## Data Availability

The data presented in this study are available upon request from the corresponding author. The data are not publicly available due to having not set up a public archive platform for data sharing.

## Supplementary Materials

The following supporting information can be downloaded at: https://www.mdpi.com/article/10.3390/metabo14060321/s1, Table S1: Primer sequence used for real-time PCR in the present study.
