# Supplementary material for: Dietary Cholest-4-en-3-one, a Cholesterol Metabolite of Gut Microbiota, Alleviates Hyperlipidemia, Hepatic Cholesterol Accumulation, and Hyperinsulinemia in Obese, Diabetic db/db Mice
Source: Metabolites. 2024 Jun 3;14(6):321. doi: 10.3390/metabo14060321 (PMC11205736; doi:10.3390/metabo14060321)
Supplement: Supplementary file 1 [file metabolites-14-00321-s001.zip › 4-STN_Supplementary files/4-STN_Editing Certificate.pdf]

Editing Certificate

This document certifies that the paper listed below has been edited to ensure that the language is clear and free of errors. The logical presentation of ideas and the structure of the paper were also checked during the editing process. The edit was performed by professional editors at Editage, a division of Cactus Communications. The intent of the author's message was not altered in any way during the editing process. The quality of the edit has been guaranteed, with the assumption that our suggested changes have been accepted and have not been further altered without the knowledge of our editors.

MANUSCRIPT TITLE

Dietary cholest-4-en-3-one, a cholesterol metabolite of gut microbiota, alleviates hyperlipidemia, hepatic cholesterol accumulation, and hyperinsulinemia in obese, diabetic db/db mice

AUTHORS

Mina Higuchi, Mai Okumura, Sarasa Mitsuta, Bungo Shirouchi

ISSUED ON

May 06, 2024

JOB CODE

BUSHI_17

Prabh Grewal
Senior Vice President - Editage

editage helping you get published

Since 2002, Editage has helped over 430,000 authors publish around 1.2 million research papers in scholarly journals across over 1000 disciplines through editorial, translation, transcription, and publication support services. Editage is a brand of Cactus Communications (cactusglobal.com), a science communication and technology company.

CACTUS.

GLOBAL :
+1(833) 979-0061 | request@editage.com

JAPAN :
0120-50-2987 | submissions@editage.com
