# Supplementary material for: Dietary Cholest-4-en-3-one, a Cholesterol Metabolite of Gut Microbiota, Alleviates Hyperlipidemia, Hepatic Cholesterol Accumulation, and Hyperinsulinemia in Obese, Diabetic db/db Mice
Source: Metabolites. 2024 Jun 3;14(6):321. doi: 10.3390/metabo14060321 (PMC11205736; doi:10.3390/metabo14060321)
Supplement: Supplementary file 1 [file metabolites-14-00321-s001.zip › 4-STN_Supplementary files/4-STN_TableS1_240509.pdf]

Dietary cholest-4-en-3-one, a cholesterol metabolite of gut microbiota, alleviates hyperlipidemia, hepatic cholesterol accumulation, and hyperinsulinemia in obese, diabetic *db/db* mice

Mina Higuchi[1], Mai Okumura[1], Sarasa Mitsuta[1], and Bungo Shirouchi[1,2,*]

[1]Nutrition Science Course, Division of Human Health Science, Graduate School of Regional Design and Creation, University of Nagasaki, Siebold, 1-1-1 Manabino, Nagayo-cho, Nishi-Sonogi-gun, Nagasaki 851-2195, Japan

[2]Department of Nutrition Science, Faculty of Nursing and Nutrition, University of Nagasaki, Siebold, 1-1-1 Manabino, Nagayo-cho, Nishi-Sonogi-gun, Nagasaki 851-2195, Japan

[*]Correspondence: bshirouchi@sun.ac.jp; Tel/fax: +81-95-813-5734

Table S1. Primer sequence used for real-time PCR in the present study.

Gene name*	Accession no.	Direction	Primer sequence (5′→3′)
Housekeeping genes			
Hprt1	NM_013556	Forward	CTGGTGAAAAGGACCTCTCGAAG
(synonym HPGRT)		Reverse	CCAGTTTCACTAATGACACAAACG
Pgk1	NM_008828	Forward	GATGCTTTCCGAGCCTCACTGT
(synonym PGK1)		Reverse	ACCAGCCTTCTGTGGCAGATTC
Rpl13a	NM_009438	Forward	CTGCTCTCAAGGTTGTTCGGCT
(synonym RPL13a)		Reverse	CCTTCCGTTTCTCCTCCAGAGT
Rpl32	NM_172086.2	Forward	GAAACTGGCGGAAACCCA
(synonym RPL13a)		Reverse	GGATCTGGCCCTTGAACCTTC
Rplp0	NM_007475.5	Forward	CCACCTGGAGAACAACCCAG
(synonym RPLP0)		Reverse	GCAGCTGGCACCTTATTGG
Tbp	NM_013684.3	Forward	GAAGAACAATCCAGACTAGCAGCA
(synonym TBP)		Reverse	CCTTATAGGGAACTTCACATCACAG
Ubc	NM_019639.4	Forward	GAGCCCAGTGTTACCACCAAG
(synonym UBC)		Reverse	CATCACACCCAAGAACAAGCA
Ywhaz	NM_011740.3	Forward	AAAGGCAGGGCGTCATTCAG
(synonym YWHAZ)		Reverse	CGATGACGTCAAACGCTTCTGG

*Gene name is represented by an approved symbol.

Hprt1, Hypoxanthine phosphoribosyltransferase 1; *Pgk1*, Phosphoglycerate kinase 1; *Rpl13a*, Ribosomal protein L13a; *Rpl32*, Ribosomal protein L32; *Rplp0*, Ribosomal protein lateral stalk subunit P0; *Tbp*, TATA box binding protein; *Ubc*, ubiquitin C; *Ywhaz,* tyrosine 3-monooxygenase/tryptophan 5-monooxygenase activation protein, zeta polypeptide.

Table S1. Primer sequence used for real-time PCR in the present study (*continued*).

Gene name*	Accession no.	Direction	Primer sequence (5′→3′)
Genes related to inflammatory response			
Ccl2	NM_011333.3	Forward	GGAATGGGTCCAGACATACATTA
(synonym MCP-1)		Reverse	TAGCTTCAGATTTACGGGTCAAC
Il-6	NM_031168.2	Forward	TACCACTTCACAAGTCGGAGGC
(synonym IL-6)		Reverse	CTGCAAGTGCATCATCGTTGTTC
Tnf	NM_013693.3	Forward	CCACGTCGTAGCAAACCAC
(synonym TNF-α)		Reverse	CCCTTGAAGAGAACCTGGGAG
Genes related to ER stress response			
Mapk8	NM_016700.4	Forward	TCTCCAGCACCCATACATCA
(synonym JNK)		Reverse	CCTCCAAATCCATTACCTCC
Xbp1	NM_001271730.1	Forward	GAGTCCGCAGCAGGTG
(synonym XBP-1)		Reverse	GTGTCAGAGTCCATGGGA
Genes related to insulin signaling and gluconeogenesis			
Akt2	NM_007434.4	Forward	CTGGGAGACCCAAGACGATAC
(synonym PKBbeta)		Reverse	CAGCATTCACACGCTGTCAC
Irs1	NM_010570.4	Forward	TGGACATCACAGCAGAATGAAGA
(synonym IRS1)		Reverse	AAGACGTGAGGTCCTGGTTG
Irs2	NM_001081212.2	Forward	TCCAGGCACTGGAGCTTTG
(synonym IRS2)		Reverse	TGGTAGCGCTTCACTCTTTCAC
Pik3ca	NM_008839.3	Forward	ACATTCCTGATCTTCCTCGTGC
(synonym PIK3CA)		Reverse	CAACGGACAGTGCTCCTCCT
Pck1	NM_011044.3	Forward	GGATGTGGCCAGGATCGAAA
(synonym PCK1)		Reverse	ATACATGGTGCGGCCTTTCA
Foxo1	NM_019739.3	Forward	GCTAAGAGTTAGTGAGCAGGCTA
(synonym FKHR)		Reverse	GGACTGCTCCTCAGTTCCTG

*Gene name is represented by an approved symbol.

Ccl2, Chemokine (C-C motif) ligand 2; *Il6*, Interleukin 6; *Tnf,* tumor necrosis factor; *Mapk8*, mitogen-activated protein kinase 8; *Xbp1,* X-box binding protein 1; *Akt2*, *thymoma viral proto-oncogene 2*; *Irs1*, Insulin receptor substrate 1; *Irs2*, Insulin receptor substrate 2; *Pik3ca*, phosphatidylinositol-4,5-bisphosphate 3-kinase catalytic subunit alpha; *Pck1*, phosphoenolpyruvate carboxykinase 1, cytosolic; *Foxo1*, forkhead box O1.
